# Violence and Capacity to Hate

**DOI:** 10.3390/healthcare11040573

**Published:** 2023-02-15

**Authors:** Giulio de Felice, Nihal Tutal

**Affiliations:** 1 Faculty of Literature and Philosophy, Sapienza University of Rome, 00100 Rome, Italy; 2 Faculty of Educational Sciences, Ankara University, 06590 Ankara, Turkey

**Keywords:** violence, hate, antisocial tendency, radicalisation, violence, psychology, group dynamics

## Abstract

This paper analyses two opposite relational configurations: violence and the capacity to hate. The former results in a psychic impoverishment, the latter in a psychic development. Primarily, the aspects of violence and the inability to hate within modern Western society are introduced. When a psychic fragility is unconsciously supported by an entire society, it becomes even more difficult to alleviate, and transform into a resource promoting psychic development. The second section explores the use of hate by young children in order to show the naturalness of this emotion and its origin. In the third and fourth sections, the unfortunate outcomes of the incapacity to hate, leading to violent antisocial conduct, are explored. To do so, the pioneering contributions by Melanie Klein and Donald Winnicott are commented on, followed by modern contributions by the literature: one of our articles published in 2020, and the review of the literature published by Alessandro Orsini on the topic of radicalisation. Finally, the differences between violence and the capacity to hate are highlighted and summarised. The article also emphasises numerous bibliographic references to further deepen the study on violence from a psycho-social perspective.

## 1. Introduction: The Failure to Recognise Hate within Modern Western Society

Modern Western society does not contemplate any space for the expression of hate and, as always happens in these cases, opposite emotions are stressed as a defensive mechanism. Indeed, we are a society based on the supposed freedom, equality and valorisation of minorities. This reaction formation [1], which involves the removal of an unpleasant emotion (i.e., hate, which is connected to fear as will be seen later), has the advantage of freeing our society from the unbearable feelings of guilt deriving from the idea of hating others, their role models, their experiences and their right to be in the world and organise their society differently from ours. In fact, those who never hate, cannot feel guilty. However, this mechanism has a side effect: the impoverishment of our society due to the lack of a possible and fruitful contamination with other types of society. Western efforts aimed at solidly maintaining the removal of hate involve a considerable waste of social and economic energy. In fact, except within the world of sport, where hate is genuinely expressed through competition and which our society allows because the sporting “game” is placed in that transitional area between fiction and reality that never affects the existing political status quo, in all other aspects of life, the emotion of hate must be rooted out. Western political forces often underline the need to fight against “the politics of hate”. Citizens’ street protests are ignored by government institutions if peaceful, but harshly condemned, and therefore rejected in toto, if there is any hint of violence within them. In the Anglo-Saxon world—a world with very strong colonialist tendencies—hate is experienced so badly that specific styles of reaction formation are implemented, which take different names based on the context of application. In academia, it is called “decolonisation of curricula”. This term indicates a government’s prescription to universities to remove from the training curricula any trace of “supremacy” of the “male and white Western world” over the rest of the world in order to ensure an equal value to the theories and scientific results of ethnic minorities. In short, decolonising university curricula means rejecting the status quo of Western (i.e., male and white; the term “Caucasian” would be too geopolitically embarrassing) hegemony of knowledge to promote alternative visions and greater academic integrity. These ideological prescriptions, which constrain educational institutions to modify their training curricula, clash with the scientific method, according to which training courses do not change due to ideologies, but rather to different scientific results. Science is perfectly egalitarian: if a Western white male says something that does not agree with the existing body of literature in that field, the content is discarded. Despite this simple basic consideration, the “decolonisation of curricula” is being carried out with the utmost force, precisely to maintain the removal of latent hate and associated feelings of guilt. Such a psychic problem can be narratively described as follows: the dyad “hate-sense of guilt” within our society is so strong and impossible to be acknowledged that the existence of differences is impeded. Assigning greater importance to Sigmund Freud, a white Western male, in the development of psychology, compared to Shirdi Sai Baba, an Indian mystic who lived at the turn of the twentieth century, is something so evident that it should not require further explanation. However, the choice to exclude the Indian mystic from the university program is associated with the history of Western colonialism and triggers the psychic alarm, which signals the existence of a very unpleasant emotional charge deriving from the combination of hate and guilt. The terror of being identified as the “white western colonialist male” is at this point so strong that, in order to avoid this psychic outcome, we decide to dedicate space in the university course on the pioneers of psychology to Shirdi Sai Baba. In the academic field, this is an example of how our society strives to maintain the repression of the hate–guilt dyad. We could expand this description by proposing examples that go beyond the academic sphere: from the accusation (once again taken from the United States) of the non-consensual nature of Prince Charming’s kiss to Snow White as an expression of male and white Western hegemony over women; to the demolition of statues of past Western explorers, whose values are judged based on modern metrics, without taking the historical–contextual factors into account. However, given that the psychic mechanism is the same and only the areas of application change, we must only add that the greater the absurdity of the accusation, the more severe the “psychopathological state” of Western society.

On the other hand, being familiar with the emotion of hate and its expression is much more normal with young children (e.g., [2]). 

## 2. Hate in Young Children

In children, hate is a natural emotion and occurs from birth. New borns hate the absence of breast milk just as they love its presence when they are hungry. In other words, they desire the proximity of the good object that satisfies their needs, and they desire distance from the frustrating object that makes their stomach cramp [2]. Therefore, the affective macro-category of love arises from the desire for closeness, the affective macro-category of hate arises from the desire for distance from the object. Both promote the identity constitution of the child and future adult. The multisensory experience of breastfeeding for a new born is the equivalent of an adult’s relationship with the world around him/her. In fact, the external world of new borns gets more and more extended over time, and this corresponds with the good growth of the subject: from the uterus to the breast, from the breast to the main caregiver, from the main caregiver to the secondary caregiver, from the secondary caregiver to the family, from the family to the social group. Therefore, the presence of the affective macro-categories of love and hate in the mind is inevitable, and characterises a good psychic development. It is the temporality that guards the birth of these two polarities. In fact, a breastfed new born will sooner or later be full, and will subsequently experience the absence of milk. The encounter between the milk of the external world and the internal need of the new born can take place with good or bad timing, and a pleasant or unpleasant affective modality. These early relational experiences, repeated over time, constitute the precursors of the relational modalities of the future adult. We have introduced the concept of affective macro-categories to indicate the two main psychic organisers of the world. The dyad love–hate or proximity–distance represents only the first affective dimension characterising the individual–external world relationship. This is a primitive affective dimension from the point of view of psychic and social development. At the neurophysiological level, it is associated with the fight–flight functioning of the brainstem, the oldest part of the human brain, and which is also possessed by reptiles. It organises the world into two categories, friend or foe, good or bad, with me or against me. There is a vast body of literature on the psychic functioning within this organisation of the individual–external world relationship (e.g., [3,4,5]). This is the relational organisation that characterises wars and strong polarisations within a social group. When there is good relational development, there is psychic growth, and within those two macro-categories increasingly complex emotions emerge, for example, embarrassment, shame, melancholy, surprise or betrayal. We can imagine affective development as a tree which, immediately after the main trunk, has two large branches corresponding to the two affective macro-categories of love and hate. Within them, as development progresses, further ramifications are formed which possess resistance equal to the frequency of the use we make of them. Mental health corresponds to a situation in which individuals do not lose the use of a wide range of possible affective resonances. They are able to feel the emotional effects that an external event has on them; therefore, they are able to live new experiences, different from those they have lived in the past. The result of this process is the expansion of the ramifications of the metaphorical affective tree. On the other hand, psychopathology corresponds to a certain degree of psychic impoverishment: the more severe the psychopathological state, the greater the psychic impoverishment. In this case, unfortunately, the individual loses the ability to use a wide range of affective resonances, because they are too painful. The outcome is the stiffening of the individual–external world relational modalities, and a progressive decrease of the ramifications of the metaphorical tree of affects [6].

We have seen how in the primary psychic development both love and hate, and their affective correlates, find space spontaneously. Both aspects are functional to the growth of the psychic apparatus in order to better manage the individual–external world relationship. How is it, then, that there can be cases in which the psychic development of hate results in violent behaviour?

## 3. Pioneering Contributions: Melanie Klein and Donald Winnicott on the Development of Antisocial Tendencies

To summarise in a single section the clinical thinking of the two most important pioneers on the subject of the development of antisocial tendencies is not an easy task. In this section the core issues are presented, but for a more detailed study the reader should refer to the original contributions.

Melanie Klein’s core contributions on this subject are: Criminal tendencies in normal children, 1931 [7]; The early development of conscience in the child, 1933 [8]; On criminality, 1934 [9]; Notes on some schizoid mechanisms, 1946 [10]; and *Envy and gratitude; a study of unconscious sources*, 1957 [11]. According to Kleinian clinical thought, antisocial behaviour is based on the death instinct, an innate drive that resides within the Id. This innatist vision of death drive, on the one hand, illustrates the need of that historical period to highlight the biological correspondent of a psychic manifestation, due to the fact that the majority of psychoanalysts at that time had medical training; on the other, it simplifies the explanation of the roots of violent behaviour. Therefore, Melanie Klein, through child clinical observation, underlines how life instinct (responsible for love and its derivatives) and death drive (responsible for hate and its derivatives) reside in the Id since birth, and how they organise a new born’s early relationships with the outside world. However, if the origin of hate and violence in childhood is quite simple in Melanie Klein’s thinking, their effects are not. Death drive distorts the infant’s parental imagos (the infant’s or child’s internal parental representations). The stronger the force of death drive, the greater the imagos will be perceived as persecutory. In other words, the more intense the death drive in the infant’s internal world, the more terrible and fearful will be its perception of caregivers. This “new born-external world” relational organisation is called by Melanie Klein “paranoid-schizoid” position. “Schizo”, because the new born’s reality is precisely “split” into two opposite polarities: friend–enemy, hate–love, me good–you bad. “Paranoid”, to underline the projective mechanism which underlies this dynamic: new borns experience an affective charge of innate hate (death drive); this is unbearable for a new born’s psyche and therefore is projected onto the main caregiver to get rid of it; the caregiver is perceived as monstrous and persecutory; new borns re-introject the figure of the caregiver who has become persecutory (depositing it within the Super-Ego); in the internal world of new borns, the fear and the need to defend themselves from such external (and internal) representations, with potentially violent behaviours, is exacerbated. The stronger the thrust of death drive, the greater the amount of hate and its derivatives to be projected onto the caregiver, the greater the persecutory nature of the internalised parental imago, and the greater the fear and need of new borns to defend themselves from such external dangers with violent behaviours. This vicious circle is brilliantly described by Melanie Klein in: The early development of conscience in the child, 1933.

Curiously, due to the hyper-specialisation of knowledge we are witnessing today, no one realises that this vicious circle is an essential foundation of the body of knowledge of geopolitics and international relations, and takes the name of “security dilemma”, published by John Hertz twenty years later [12]. Briefly, the “security dilemma” is based on the fear that one “A” state might be attacked by another “B” state. In this case “A”, as a precaution, may decide to expand its internal and external security. This behaviour is read by “B” as a clear indication of “A”s’ intention to attack “B”. State “B” then decides to expand its internal and external security as well. Hence, “A” and “B” continue to raise their security level until they come to a direct confrontation. Over time, the literature on “security dilemma” has taken various variables into consideration, among which, one of the most important, is greediness (e.g., [13,14]). For the sake of clarity and brevity, it is left to interested readers to deepen their knowledge of this connection with geopolitical dynamics.

Within this paranoid–schizoid organisation, a new element now takes over for the new born: the realisation that the outside world is not only persecutory and monstrous, but it also supplies good milk that provides nourishment and generates a psychophysiological condition of satisfaction and contentment. This evidence, which makes its way into the new born’s psyche with each episode of breastfeeding, in the good psychic development, breaks the vicious circle described above. Thus, according to the Kleinian clinical description, the new born now enters the “depressive” position. “Depressive”, precisely because he/she realises that it has attacked the external object that provides he/she with care and sustenance. This is how the first feelings of guilt arise, which for Melanie Klein constitute the precursors of the child’s prosocial behaviours. In this “position”, Klein observes that the way a child plays usually expresses reparative tendencies, such as trying to glue a previously broken pencil back together; new borns then display an ever-growing desire to be loved, to love and to be at peace with the world around them; a more real and less monstrous perception of caregivers; a reduction of fear in their internal world; a renewed confidence in the outside world.

Donald Winnicott’s contributions on the subject have been collected in the second part of the book entitled, *Deprivation and Delinquency*, 2015 [15]. In full, the main ones are: Aggression and Its Roots, 1964; The Development of the Capacity for Concern, 1963; The Antisocial Tendency, 1956; Youth Will Not Sleep, 1964. The origin of antisocial tendencies, for Donald Winnicott, is to be linked to an experience of “true deprivation”. By “true deprivation”, Winnicott means an experience of loss that a child has experienced: a positive relationship that has broken, and of which there is no longer a trace in conscious memory. True deprivation, therefore, implies two different aspects: the first is linked to the traumatic experience itself (loss), the second is linked to the consecutive condition of deprivation which sustains the trauma over time. In the worst-case scenario, this gives rise to the complete loss of hope of finding the lost good object. In other words, the good object, the original emotional sustenance for the individual, has not only been lost, but the condition of loss continues over time, generating deprivation. In the 1950s, the link between deprivation and antisocial tendencies was also studied by John Bowlby [16], a British psychoanalyst who greatly influenced Winnicott on this topic. Therefore, antisocial violence, according to Winnicott, is interpreted as a behaviour that conveys the hope of finding a good object in the environment capable of providing for the emotional sustenance of the subject. Furthermore, antisocial behaviour involves a highly disturbing aspect for the environment. Winnicott values this last characteristic of antisocial conduct (i.e., nuisance) because it forces the environment to deal with violent subjects and register what they do, in the hope that it will be capable of meeting their emotional needs. In fact, antisocial people are looking for an environment that resists their aggression, and takes care of their emotional needs.

The origin of antisocial tendencies has been shown to differ according to the pioneering contributions of Melanie Klein and Donald Winnicott. The first connects its genesis to death drive, an innate unconscious force lying in the Id. The second connects it to an experience of true deprivation, in which the child has lost the good object that previously provided for his/her emotional needs. These two different perspectives are transformed into two different therapeutic positions. Melanie Klein underlines the importance of addressing the aggressive aspects present in the patients’ internal world in order to alleviate them, making their expression and recognition possible within the therapeutic session. Donald Winnicott underlines the importance of addressing the patient’s experience of loss and consequent deprivation, identifying the therapist’s role as the auxiliary good object catering for the patient’s needs. The aim of this therapeutic stance is for the patient to re-introject the function of emotional support performed by the therapist during treatment. Hence, to eliminate antisocial behaviours means, for Melanie Klein, to alleviate the persecutory nature of the patient’s internal world, whereas for Winnicott, it is to provide the correct sustenance of the patient’s deprived Ego. Aggression and deprivation are still the two clinical pillars with which the literature gives meaning to violence in antisocial patients (e.g., [17]).

## 4. Modern Contributions on Antisocial Tendencies, from Normality to Criminality

In our article, “A psychoanalytic contribution to the understanding of criminal tendencies”, 2020 [18], we bring to the attention of the scientific community further elements underlying violent behaviour. First, we introduce the notion of “psychic vitality”. Psychic vitality can derive from both the affective macro-categories of love and hate. When it comes from love, we can call it “+ psychic vitality”; when it comes from hate “− psychic vitality”. Examples of positive psychic vitality are the prosocial behaviours of love, tenderness, gratitude; they help others and are of emotional nourishment for the psyche as they are re-introjected into the Ego. Examples of negative psychic vitality are the possible positive transformations of hate, sadness, aggression and violence. These can become, for example, assertiveness, or introspective melancholy, and help to achieve an important goal. Therefore, the opposite of psychic vitality is not “− psychic vitality”, as important as “+ psychic vitality”, but “*psychic dryness*”. This is defined as that condition of extreme psychic impoverishment in which the individual no longer finds any pleasure in relating to the outside world. In this case, which unfortunately corresponds to very severe psychopathological conditions, the individual–external world relationship is completely interrupted. The individual loses the ability to process external reality in psychic terms. The condition of psychic dryness, by cutting ties with the outside world, represents the most primitive configuration of the individual–outside world relationship, in which development and mutual emotional enrichment are totally precluded. The individual–external world configuration that immediately follows is characterised by an undifferentiation of psychic vitality. We have called the functioning of the mind within this configuration “*autonomic-like functioning*”. In this type of organisation, the mind regains its ties with the outside world. However, these links can only be turned on or off. There are no intermediate emotional shades. There is an activation of the individual–external world interaction or its deactivation. Within this psychic organisation, the mind behaves in a similar way to the autonomic nervous system. On the one hand, there is the possibility to “rest” and “digest”, the “parasympathetic” pathway which drives the organism to have no relationship with external objects. On the other hand, there is the possibility of “fight” and “flight”, the “sympathetic” way that drives the organism towards object relations. In fact, both these latest behaviours require an external object with which the organism is engaged. If the sympathetic pathway is activated, there are no further affective differentiations in the individual–external world relationship: fighting-dominating-destroying (“− psychic vitality”) and loving-sharing-caring (“+ psychic vitality”) mean the same thing. Finally, the most mature configuration of the individual–external world relationship is the one in which positive and negative psychic vitality are differentiated (“*differentiation of psychic vitality*”). In this latter case, the mind manages to understand that fighting-dominating-destroying and loving-sharing-caring are two distinct ways of object relation, which produce different affective outcomes both in the object and in the subject itself. We have proposed antisocial violence as a behaviour pertaining to the second configuration of the individual–external world relationship (i.e., autonomic-like functioning), in which there are no differences between positive and negative psychic vitality. Antisocial violence, in these cases, means exhibiting the behaviours of fighting-dominating-destroying and loving-sharing-caring at the same time.

The creation of new identity meanings in the process of violent radicalisation of individuals who then commit criminal acts is a core issue underlined by the world’s leading experts of this field. For an accurate review of the literature, we recommend the article by Alessandro Orsini, “What everybody should know about radicalization and the DRIA model”, 2020 (i.e., Disintegration, Reconstruction, Integration, Alienation) [19]. The author compares the most important theoretical models on the genesis and development of radicalisation of subjects who then commit acts of terrorism. In fact, he takes into consideration the contributions of the following international researchers: Fathali M. Moghaddam, Silber and Bhatt, Marc Sageman, John Horgan, Quintan Wiktorowicz, Lawrence Kuznar, Clark McCauley, Sophia Moskalenko, Donatella della Porta, Arie W. Kruglanski, Jocelyn J. Bélanger and Rohan Gunaratna. Among the most significant for the present discussion are the contributions of Fathali M. Moghaddam, which led to the establishment of his “staircase to terrorism”. This author theorises a scale with, at each step, specific psychological and social conditions of the individual. In the first step, there is a large part of the existing population, in the last step only radicalised individuals who commit terrorist acts. In particular, in the first step of this scale, the author places individuals who would like to improve their social status, and who feel they have suffered a relative deprivation. They see what others are achieving and feel that they have not achieved what they deserve. The second step includes individuals who want to seek a remedy for the injustices they feel they have suffered. If they perceive the society in which they live as one organised in such a way as to prevent the improvement of their social status, and therefore as a dishonest society, the chances of climbing to the third step are high. In the third step, due to the impossibility of responding aggressively towards the source of frustration (it is impossible to change the whole society with the efforts of a single individual), the individuals move their aggression outside, creating a polarised friend–foe situation. In the fourth step, individuals perceive the actions carried out by terrorist organisations as the only way to reform society and socially redeem themselves. In other words, in this stage the moral coordinates of individuals increasingly coincide with those of terrorist organisations, which are perceived as the only social group capable of understanding them. In the fifth step, individuals have now become part of terrorist groups and cannot get out alive. In this stage the polarised friend–foe worldview is consolidated. The values of these people are identified with those of terrorist groups. Their identity is now transformed, and violence is perceived as necessary. Finally, the individuals of the sixth step carry out terrorist acts, and do not perceive any feeling of guilt towards their victims, as they perceive them to be the corrupt enemy which must be killed. Therefore, the path to terrorism, according to Fathali M. Moghaddam, derives from the mismatch between what an individual or group think they deserve, and what they think they can really achieve. A society that promotes citizens’ involvement and esteem acts as a significant protective factor against the development of this type of criminal conduct.

The second outstanding contribution within the review we are discussing in this section is by Alessandro Orsini. He calls his theoretical model on radicalisation the DRIA model. This model summarises the various contributions of the literature, including both the scientific contributions of scholars who assign a decisive importance to the social group in fostering individual radicalisation, and the contribution of scholars who instead assign greater importance to ideology. To expand further the acronym, DRIA stands for: Disintegration of social identity; Reconstruction of social identity through a radical ideology; Integration into a revolutionary sect; Alienation from the surrounding world. The first stage is characterised by the disintegration of the individual’s social identity, who goes through an existential crisis. For instance, traumatic events can undermine an individual’s social identity. At this stage the individual feels that, in the society in which he/she lives, there is no place for the values in which he/she believes. In a psychic and social situation of this kind, embracing a radical ideology is one of the ways that the individual can choose to rebuild his/her social identity, and give meaning to his/her existence. Those who choose this type of solution enter the second stage, in which the individual’s social identity is rebuilt on the basis of a radical ideology. For people who reach this stage, the world appears divided in two opposite poles: friends on one side and enemies, who are not viewed as human beings but only symbols to be attacked, on the other. Individuals in the third stage, after embracing the radical ideology, begin to look for groups of people who share the same beliefs. They form a radical group, real or imaginary. Finally, in the fourth stage, there is the complete separation, concrete and ideological, of the radicalised group from the surrounding world. This clear demarcation serves the identity of the group, which, at this point, does not interact with any alternative model of existence.

To summarise, the contributions of researchers on radicalisation processes highlight three common fundamental pillars [20]. The three Ns of radicalisation are: Needs, Narratives and Networks. The basic narcissistic Needs, when constantly frustrated, generate a deep feeling of self-devaluation and of being an outcast from society, with the associated profound difficulty of giving meaning to one’s existence. In this situation, the Narratives of terrorist groups hook up with the narcissistic need of the subject, providing a potential way out of the previous, merciless, psychological condition. The Network of the terrorist group restructures the individual’s identity, giving rise to the well-studied relational dynamics of fight–flight, good–bad or friend–foe. Once identified with this psychic configuration of the world, the individual can do nothing but engage in war, of which terrorist acts and crimes are a part.

## 5. Conclusions: Violence vs. Capacity to Hate

Violence represents only one of the possible individual–external world relationships. In the previous sections, many elements underlying the violent relationship have been highlighted: the lack of recognition of hate in modern Western society; the link between violence, hate–fear (Klein) and deprivation (Winnicott); the undifferentiation of meanings present in some violent behaviours (autonomic-like functioning); the role of ideology and social groups in the violent radicalisation of the individual (Table 1).

At the basis of the violent individual–external world relationship, there is always the fight–flight psychic configuration that, as we have mentioned, polarises the world into friend–foe or good–bad. This psychic configuration implies a serious side effect: the denial of “otherness”. The others, those who are different, with their needs, their emotions, their right to be in the world and actively participate in everyday life, simply do not exist. They end up in the psychic macro-category of the “enemy” who must be attacked, or from whom one must flee. The specificity of this psychic macro-category lies in the fact that the attributes of the “enemy” do not derive at all from knowing the enemy, but are the result of splits and projections of the subject, who, therefore, sees in others that which they just do not tolerate in themselves. Consequently, in this case nothing new is ever learned from the world and from the succession of events. There is only a constant repetition of the same relational dynamic applied to different external objects, which, for the subject, never differ; indeed, they never possess an existence of their own. They only exist as more or less suitable “coat hangers” for the subject’s projections.

The effects of the fight–flight psychic configuration were also evident in the public health arena. In November 2021, we compared COVID-19 vaccines distribution with the existing geopolitical relationships. It is sad to admit that health devices made to save people’s lives were not distributed based on the effectiveness shown in scientific studies but based on geopolitical influences [14]. This result showed the power of such psychic configuration, which prevailed even during a pandemic, in which one would expect a genuine cooperation between different nations.

Moreover, there is an even worse side effect of the individual–external world relationship discussed here; that of the psychic structure being passed down from generation to generation. Therefore, a family totally permeated by this psychic impoverishment, infects its own children, so that they will have numerous difficulties in emancipating themselves from this internal structure. A society totally permeated by this psychic impoverishment infects the next generation. These new generations will then perceive different societies, with their own models and experiences, as external objects to be demolished, conquered, or shunned. In fact, learning something new from the outside world implies recognition of “otherness”, recognition of the differences of such an external world with respect to the subject, a characteristic which the friend–foe structure is totally lacking. On the other hand, such a characteristic is at the basis of the capacity to hate.

Within this other psychic configuration there is, therefore, the full recognition of diversity, which is of nourishment for the object and for the subject. In the child, the first verbal manifestation of the affective macro-category of hate is the word “no”. Which means “you are different from me”, “that thing is not part of me”. Identity could not exist except as a result of the continuous and fluctuating desires of proximity (identification) and distance (expulsion) from external objects. Therefore, the capacity to hate implies a serene acceptance of the affective macro-category of hate within our internal world, within our institutions, within our society. To acquire such capacity, the person, the institution, the society must learn to competently hate. If hate is not associated with knowledge, it becomes a preconceived judgment, and with it, we enter the friend–foe psychic configuration, which has been widely discussed. To competently hate, there is a need to know the “others”, to understand their values and personal feelings, to understand their differences; to avoid assuming that our standards are recommendable to others; to avoid exporting the models with which we see the world. Our society has enough fragilities to allow us to learn from others. A wonderful opportunity presents itself: to be able to hate in order to know, grow and be enriched through the relationship with diversity.

## Figures and Tables

**Table 1:** Different characteristics underlying violence and the capacity to hate.

Violence	Capacity to Hate
Lack of recognition of hate in society	Recognition of hate in society
Vicious circle hate–fear in the individual–external world relationship	Virtuous circle love–trust in the individual–external world relationship
True deprivation	Absence of deprivation
Undifferentiation of the meanings of violence	Differentiation of positive and negative psychic vitality
Three Ns of radicalisation	Sustenance of individuals’ basic narcissistic needs

## Data Availability

Data are available, Data supporting the findings and conclusions are available upon request from corresponding author.
